# Morphologic and chemical composition of particulate matter in motorcycle engine exhaust

**DOI:** 10.1016/j.toxrep.2018.01.003

**Published:** 2018-02-02

**Authors:** V.V. Chernyshev, A.M. Zakharenko, S.M. Ugay, T.T. Hien, L.H. Hai, A.S. Kholodov, T.I. Burykina, A.K. Stratidakis, Ya. O. Mezhuev, A.M. Tsatsakis, K.S. Golokhvast

**Affiliations:** a Far Eastern Federal University, Sukhanova Street, 8, Vladivostok, 690950, Russian Federation; b Laboratory of Toxicology, School of Medicine, University of Crete, Heraklion, 71003, Greece; c Dmitry Mendeleev University of Chemical Technology of Russia, Miusskaya Square, 9, Moscow, 125047, Russian Federation; d Pacific Geographical Institute FEB RAS, Vladivostok, Russian Federation; e Department of Analytical Toxicology, Pharmaceutical Chemistry and Pharmacognosy, Sechenov University, 119991, Moscow, Russian Federation

**Keywords:** PAHs, polycyclic aromatic hydrocarbons, ICE, internal combustion engines, EDTA, ethylenediaminetetraacetic acid, EFI, electronic fuel injection system, PM, particulate matter, PM_10_, particles with a diameter between 2,5 and 10 μm, VEPs, vehicle emitted particles, VOCs, volatile organic compounds, Polycyclic aromatic hydrocarbons, Particle size distribution, Chemical composition, Motorcycle exhaust gases, Environmental toxicology

## Abstract

Despite the fact that environmental pollution due to motorcycle exhaust gases reports a great increase, motorcycle production exhibits a great increase through the last years. Countries of Asia and Africa are reported to be the major regions where two-wheeled vehicles are a major transportation mode, with tens of millions of units sold per year. Motorcycle exhaust particles are considered to be the major contributor to environmental pollution due to their airborne dispersion, containing great amount of polycyclic aromatic hydrocarbons (PAHs). This study aims at reporting an objective analysis of the main sources of the ambient air pollution as also particle size distribution and chemical composition analysis of particulate matter originated from the exhausts of two-wheeled vehicles used in the territory of Vladivostok, Russia. Various types of two-wheeled vehicles were examined (motorcycles, ATVs, scooters and wet bikes) using different types of engine and fuel system. Experimental results showed that there was no clear relation to the particle size distribution with the engine displacement of motorcycle and the number of strokes and the fuel system. Instead, there were reported two clear assumptions. The first one is that regarding to the motorcycle brand, a few samples did not exhibit a great percentage of PM_10_ fraction. The second one is that more modern vehicles, that have a harmful gas afterburning system, are usually the source of an increased percentage of PM_10_ emitted particles. At last, it should be mentioned that the laser particle size analysis method is capable of determining the particle sizes after their agglomeration whereas the optical morphometry method allows to determine the real particle size of emissions. In conclusion, it can be pointed out that the agglomeration of particles can lead to the reduction in the toxicity of particles emissions originated from two wheeled-vehicles.

## Highlights

• Regarding to the motorcycle brand, a few samples did not exhibit a great percentage of PM_10_ fraction.
• Modern vehicles are usually the source of an increased percentage of PM_10_ emitted particles.
• Particles under PM_2,5_ fraction cause micro climatic effects by absorbing the sunlight.
• Laser particle size analysis method is capable of determining the particle sizes after their agglomeration.
• Particle size distribution and engine displacement do not exhibit a strong relationship.

## Graphical abstract

**Figure:**
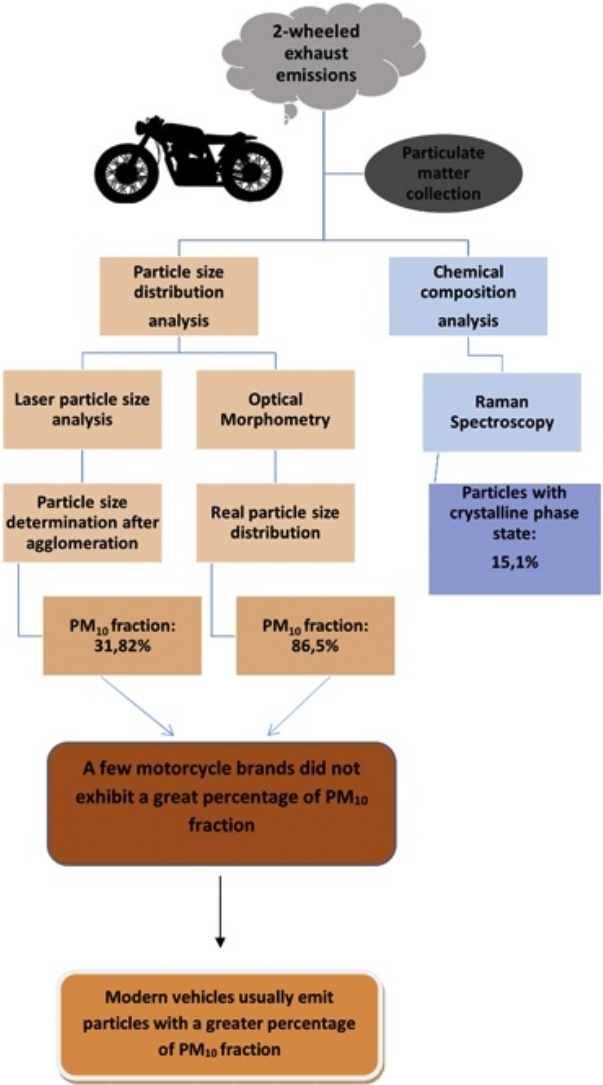

## 1 Introduction

Polycyclic aromatic hydrocarbons (PAHs) are a group of hazardous chemicals, toxic to human health [[1], [2], [3]] while also considered as potent atmospheric pollutants when found in the environment, as many of their compounds have been identified as carcinogenic, mutagenic, and teratogenic to microorganisms [[4], [5], [6], [7]]. According to previously research studies it is reported that due to their high levels of lipophilicity and water-insolubility it is difficult to be degraded by microorganisms [8] which makes their presence in the environment even longer. In urban atmosphere, PAHs are mainly anthropogenic, originated from incomplete combustion of fuels in the engines of transportation means.

According to the literature, various types of vehicles have been examined concerning their pollutant emissions [[9], [10], [11], [12]] where motorcycles and diesel powered cars have proved to be the main sources of maximum emissions of particulate matter, while in many countries it is reported that the major polluting factor of ambient air comes from the emissions of motorcycles, comparing to all transportation modes [13]. Despite the small engine displacement of two-wheeled vehicles, comparing to automobiles, the particulate matter originated from motorcycles’ exhaust gases is reported to be greater in amount than PM originated from automobiles while also exhibited stronger PAH-related carcinogenicity and indirect mutagenicity than PM from automobiles [14]. The microscale pollution of the ambient air by human sources, caused by internal combustion engines (ICE), has been actively studied in recent years due to their dominant contribution to the environmental [[11], [12], [14], [15]]. More precisely, the largest contribution is made by the dispersion of micro and nanoscale fractions of the air pollutant emissions of cars and motorcycles [[7], [16]].

Two-wheeled vehicle transportation is one of the most important transportation means in Russia. On a global scale, the most major regions were motorcycles are used are countries of Asian and African continents, with tens of millions of units sold per year. The production of motorcycles in these countries is growing much faster than the production of automobiles, while since 2003 the global production of motorcycles has increased 42%, 75% of which was reported in Asian countries by 2006, while it continues to grow [17]. Pollution of ambient air in nano and micro scale is also a matter of great importance in cities of Russia’s Far East. Vladivostok and Ussuriisk are reported to be the cities with the greatest percentages of vehicles used as it is estimated that the ratio of the total number of cars and motorcycles to the total population exceeds 60% [[7], [18]]. In these cities the contribution of exhaust gases of motorcycles to the pollution of the ambient air is quite high. Despite the fact that two-wheeled transportation in the Far Eastern region is seasonal, the number of motorcycles and scooters in the spring-autumn period in residential and adjacent territories counts to a significant number.

Previously reported studies [[18], [19], [20]] have shown that it is possible to estimate the environmental hazard degree of a specific vehicle with in-depth analysis of exhaust emissions of ICE. Specifically, in the exhaust gases of two-wheeled vehicles, an increased content of PAHs and regulated air pollutants (CO, CO_2_, HC, NO_x_, PM) is found with a wide particle size range of particulate matter [[10], [15], [21]]. Till today a great number of research studies have investigated the particle size distribution of vehicle emitted PM and their chemical composition [[22], [23], [24], [25]]. As reported, the toxic effects of particulate matter to human health are strongly connected to the size of particles emitted and their chemical composition. As shown in the past, exhaust emitted particles sized under the PM_10_ and PM_2,5_ fractions can cause various toxicological effects [[26], [27]]. These toxicological effects is also proved to vary according to the chemical composition of PM [28].

Regarding to the type of engine, 2-stroke or 4-stroke, and the type of fuel system used, carburetor or EFI, there have been reported some differences in the amount of emitted exhaust gases. More specifically it has been reported that motorcycles using the EFI fuel system emit lower amount of air pollutant particles than motorcycles using carburetor fuel systems [15], which leads to the conclusion that two-wheeled vehicles using the EFI fuel system are environmentally safer than those using the carburetor. In relevant studies it has been reported that particles emitted from 2-stroke engines tend to be more toxic than 4-stroke engines, as 2-stroke engines exhibit the disadvantages of incomplete combustion and less efficiency due to mixed combustion of engine oil and gasoline [11]. Two-stroke scooters have been reported to be a major source of air pollution in many cities [29], while there are scientific suggestions that scooters will emit more PAHs than all other vehicles combined in Europe by 2020 [30]. Due to this fact, TEPA (Taiwan Environmental Administration Protection), strongly encourages the use of 4-stroke engines rather than 2-stroke engines, aiming this way to proceed to new environmental regulations [31].

Considering all the above, the need for further research concerning the toxic effects of PAHs originated from exhaust emissions of two-wheeled vehicles to human health and environment seems to be imperative. This study is dedicated to the estimation of human health and environmental risks produced by toxic exhaust gas emissions of two-wheeled transportation means. For this reason an objective analysis of the main sources of the ambient air pollution was carried out as also complete investigation of the particle size distribution and chemical composition of particulate matter originated from exhaust emissions directly from their source in commercially available models of motorcycles, ATVs, scooters and wet bikes used in the region of Vladivostok, Russia.

## 2 Materials and methods

In this study 44 different two-vehicle models (Table 1), using different engine types, fuel systems and different displacement, were examined. Samples of particulate matter in the exhaust emissions from engines of all vehicles were collected as experimental data (Table 1).

**Table 1:** List of motorcycles, ATVs, scooters and wet bikes used in the experiment.

No	Coded vehicle model	Displacement(cc)	Year of manufacture	Fuel (Russian standard)	Engine type	Fuel system	Mileage (km)
1	HCB	400	2002	Gasoline AI-95	4-stroke	Carburetor	17700
2	HCRF	450	2005	Gasoline AI-95	4-stroke	Carburetor	130 moto hours
3	HCRF	250	2005	Gasoline AI-95	4-stroke	Carburetor	200 moto hours
4	YYZF	250	2008	Gasoline AI-95	4-stroke	Carburetor	180 moto hours
5	HXr	100	2005	Gasoline AI-95	4-stroke	Carburetor	23000
6	KKXF	450	2008	Gasoline AI-98	4-stroke	Carburetor	120 moto hours
7	SVS	1400	1988	Gasoline AI-92	4-stroke	Carburetor	40000
8	SGSF	400	1992	Gasoline AI-92	4-stroke	Carburetor	80000
9	SGSF	600	1992	Gasoline AI-92	4-stroke	Carburetor	75000
10	SGSXR	1000	1995	Gasoline AI-92	4-stroke	Carburetor	35000
11	YR	1500	1997	Gasoline AI-92	4-stroke	Carburetor	30000
12	HXr	250	1997	Gasoline AI-92	4-stroke	Carburetor	7500
13	KZZR	400	1997	Gasoline AI-92	4-stroke	Carburetor	50000
14	HCB	1300	1998	Gasoline AI-92	4-stroke	Carburetor	30000
15	YGP	1200	2000	Gasoline AI-92	4-stroke	Carburetor	30000
16	YRZ	760	2000	Gasoline AI-92	4-stroke	Carburetor	30500
17	HCB	400	2000	Gasoline AI-92	4-stroke	Carburetor	14514
18	YXjr	1300	2000	Gasoline AI-92	4-stroke	Carburetor	38000
19	YFZS	600	2001	Gasoline AI-92	4-stroke	Carburetor	41000
20	HCB	400	2001	Gasoline AI-92	4-stroke	Carburetor	39000
21	SB	800	2002	Gasoline AI-92	4-stroke	Carburetor	45000
22	HCB	1300	2003	Gasoline AI-92	4-stroke	Carburetor	40000
23	HCB	1300	2005	Gasoline AI-92	4-stroke	Carburetor	35000
24	YYZF	450	2005	Gasoline AI-92	4-stroke	Carburetor	200 moto hours
25	Ksxf	350	2011	Gasoline AI-98	4-stroke	EFI	30moto hours
26	BRPSD	1500	2009	Gasoline AI-95	4-stroke	EFI	150moto hours
27	Ksxf	350	2012	Gasoline AI-95	4-stroke	EFI	100moto hours
28	KU	1500	2012	Gasoline AI-95	4-stroke	EFI	100 moto hours
29	HCBRXX	1000	2003	Gasoline AI-92	4-stroke	EFI	37000
30	SB	1800	2004	Gasoline AI-92	4-stroke	EFI	50000
31	YFJR	1300	2005	Gasoline AI-92	4-stroke	EFI	25000
32	AC	700	2005	Diesel	4-stroke	EFI	140000
33	K	700	2007	Gasoline AI-92	4-stroke	EFI	50moto hours
34	BRPSD	1800	2009	Gasoline AI-92	4-stroke	EFI	28000
35	KU	1400	2009	Gasoline AI-92	4-stroke	EFI	10200
36	YFZ	1800	2010	Gasoline AI-92	4-stroke	EFI	200 moto hours
37	BRPSD	1800	2011	Gasoline AI-92	4-stroke	EFI	220 moto hours
38	KS	1500	2011	Gasoline AI-92	4-stroke	EFI	85moto hours
39	HD	50	1991	Gasoline AI–92 + oil	2-stroke	Carburetor	3100
40	HCR	125	1991	Gasoline AI–92 + oil	2-stroke	Carburetor	180 moto hours
41	HCr	80	1992	Gasoline AI–92 + oil	2-stroke	Carburetor	100 moto hours
42	HCRM	250	1997	Gasoline AI–92 + oil	2-stroke	Carburetor	250 moto hours
43	KSX	250	2012	Gasoline AI–92 + oil	2-stroke	Carburetor	150 moto hours
44	KSX	125	2010	Gasoline AI–95 + oil	2-stroke	Carburetor	100 moto hours

### 2.1 Sampling methods for the particles from vehicle exhaust emissions

The samples were removed using a scraper by collecting soot (crystalline structure) or ash (amorphous structure) from the exhaust manifold of motorcycles, placed in a sterile container, and then experimental measurements were carried out. All samples were treated as described earlier for the cars particles exhaust [18] and then sent to the laboratory for further research.

The samples were collected as described earlier for the cars particles exhaust [18] and then sent to the laboratory for further research.

### 2.2 Characterization of particulate matter from exhaust emissions

The histograms of particle size distribution for samples of PM were determined by Laser particle size analysis using laser particle sizer ANALYSETTE 22 NanoTec plus (Fritsch GmbH, Germany). Samples were dissolved under ultrasound and with the addition of specialized surfactants (EDTA) and ethyl alcohol.

In addition, the histograms of particle size distribution of PM were determined by Optical morphometry with Raman spectroscopy using the automated Raman microspectroscopy system G3SE-ID (Malvern Instruments Ltd., UK). For the examination of the samples 10^5^ particles from each sample were analyzed on average.

Identification of the phase state of carbon and classification in crystalline or amorphous type as also its fraction in the sample, using the Raman Rxn1™ Analyzer (Kaiser Optical Systems, Inc.) was carried out with the combined light scattering method, and 10^3^ particles were analyzed on average.

## 3 Results

**Fig. 1:**
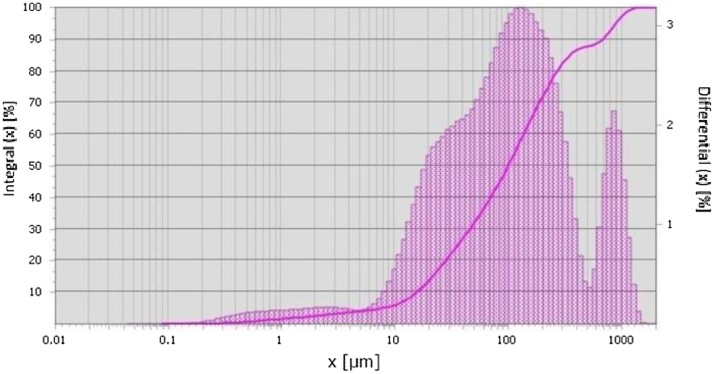
Particle size distribution of sample 1 measured with laser light scattering.

According to the results obtained by laser particle size analysis for sample 1, it was found that particle size distribution exhibits an increase in the percentages of particles sized over the PM_10_ fraction (Fig. 1).

**Fig. 2:**
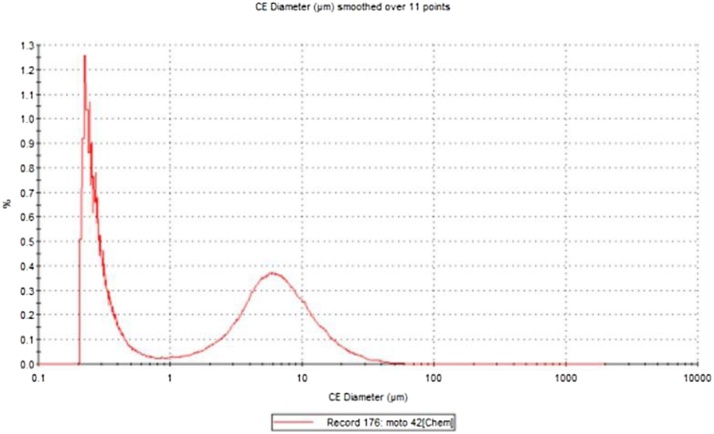
Particle size distribution of sample 1 measured with optical morphometry.

As can be seen in Fig. 1, only large particles, or agglomerates, are identified while the percentage of particles sized under the PM_10_ fraction is 5.4%. For the verification of the results obtained bylaser particle size analysis, the same dry sample using was measured using optical morphometry (Fig. 2).

According to the results from optical morphometry it is shown that the percentage of particles sized under the PM_10_ fraction is 68,69%.

All results of the measurements of all samples listed in Table 1, with both methods of particle size analysis and optical morphometry are presented in Table 2.

**Table 2:** Listed results of particle size distribution and chemical composition of samples.

No.	PM_10_ fraction using laser particle size analysis (%)	PM_10_ fraction using optical morphometry (%)	Fraction of particles with structured carbon (crystalline phase state), (%)
1	5.4	68.7	62.6
2	22.4	67.4	1.4
3	27.7	98.7	100.0
4	29.6	84.7	3.1
5	33.5	93.1	14.3
6	23.8	95.0	12.5
7	43.1	99.4	53.9
8	31.5	98.9	2.6
9	34.5	95.5	4.0
10	32	95.5	13.3
11	13	89.9	8.8
12	29.7	75.7	0
13	17.7	93.4	1.7
14	64.8	97.4	63.2
15	36.7	58.8	2.0
16	14	84.0	0.7
17	19.7	73.0	9.0
18	16.6	61.5	0.6
19	22.6	95.3	1.8
20	28.1	97.6	2.2
21	14.5	49.3	0.1
22	55.1	94.5	0
23	18.5	91.3	0
24	39.7	85.6	7.4
25	39.5	91.6	11.8
26	28.6	81.6	1.8
27	37.2	94.3	0.6
28	19.1	57.6	3.6
29	25.9	79.00	7.5
30	47.5	97.0	35.9
31	32	91.7	42.8
32	24.6	76.1	2.9
33	50.9	98.8	19.7
34	30.9	76.7	6.6
35	15.3	86.5	2.3
36	28.6	93.0	1.0
37	25.8	93.5	2.1
38	30	78.5	6.7
39	64.4	93.7	5.2
40	24.2	84.6	12.5
41	20.6	99.6	14.3
42	19.4	97.9	20.5
43	71.7	92.3	58.1
44	20.8	98.2	28.7
Median	31.82	86.5	15.1

As shown in Table 2, when optical morphometry is used, particles sized under the PM_10_ fraction, which are the smallest and therefore the most hazardous to human health, are dominant among other fractions. In 25 samples out of 44 the content of PM_10_ particles is over 90% while in 43 samples is over 50%.

## 4 Discussion

Experimental results of particle size distribution, listed in Table 2, exhibited great differences when performed by the two methods of characterization. As shown in particle size distribution determined by laser particle size analysis, the major percentage of PM identified is consisted mainly of large particles and agglomerates, while the percentage of particles sized under the PM_10_ fraction is 30.25%. Only for 4 samples the PM_10_ fraction is exhibited to be over 50%. For all 6 samples using 2-stroke types of engines the PM_10_ fraction is 36.8% comparing to the other 38 samples, using 4-stroke types of engines, in which the percentage of PM_10_ fraction is 29.21%. Only for samples 14, 22, 33, 39 and 43 the amount of PM_10_ particles is more than 50%. There was no clear relation to the particle size distribution with the engine displacement of each vehicle as also with the number of strokes and the fuel system. Instead, there were reported two clear assumptions. As shown, some brands did not seem to emit great amounts of PM_10_ fraction when examined by both methods of characterization, as also more modern vehicles that have a system for afterburning harmful gases are usually a source of an increased content of particles PM_10_ fraction.

The results of particle size distribution obtained by optical morphometry correlate with the results of studies conducted in China, as it was shown that the arithmetic mean diameter of 90.3% of the exhaust particulate matter from two-stroke scooters with 50 cc displacement is in the range of 0.1–1 μm [12]. According to the results, in 25 samples the percentage of particles sized under the PM_10_ fraction is reported to be 86.50%, while only for 1 sample is reported to be under 50%. For all 6 samples using 2-stroke engines the PM_10_ fraction is 94.39% while for all 38 samples PM_10_ fraction is 85.25%. In this case, the particle size distribution mainly depends on the number of strokes of the engine rather than the engine displacement.

The method of laser particle size analysis allows us to determine the particle size distribution after partial agglomeration, which differs from the initial particle size distribution, determined by the method of optical morphometry. Apparently, this peculiarity is more obvious when 2-stroke vehicles are examined. This can be explained by the design features of two-stroke internal combustion engines as particles in the exhaust contain greater amounts of oil, which makes them more hydrophobic. As a result, particles tend to aggregate, and their agglomerates cannot be dissolved even under ultrasound. Unfortunately, the addition of specialized surfactants (EDTA) and ethyl alcohol is not able to improve the results. On the contrary, when optical morphology is used, all samples are firstly dried, leading to the removal of oil. In that case, particles did not form aggregates and the PM_10_ fraction was more notable.

In addition, it is worth noting that more modern engines are in pursuit of reducing the amount of exhaust gases, but the catalysts used for this lead to a significant increase of the PM_10_ fraction percentage. This fact explains why in one of the most modern engines, manufactured in 2012 (sample 43), the greatest percentage of PM_10_ fraction was found. Modern engines succeed in reducing the exhaust gases, but they as well emit particles sized under the PM_10_, which are more hazardous to the environment.

Size distribution of emitted particulate matter from exhaust gases of two-wheeled vehicles is strongly related to toxic effects to human health and environmental pollution. Presence of agglomerates in the total mass of PM induces reduction in the surface/mass ratio of particles, and thus their capacity to absorb organic compounds. This fact leads to the reduction of their toxicity to human health and environment [32]. A great amount of previously reported studies have identified a strong connection between inhalable particles distributed in the PM_2.5_ and PM_10_ fractions and a wide range of health effects related to cancer, allergies, asthma and lung inflammation, cardiovascular diseases and respiratory diseases [[33], [34], [35], [36]]. It is also well known that particles sized in the PM_10_ and PM_2.5_ fractions are capable of entering the respiratory system and can potentially be deposited anywhere within the respiratory tract [[37], [38]]. Considering all the above, it is clear that the presence of large particles and agglomerates is a crucial factor that can lead to reduced toxicity of exhaust gases.

**Fig. 3:**
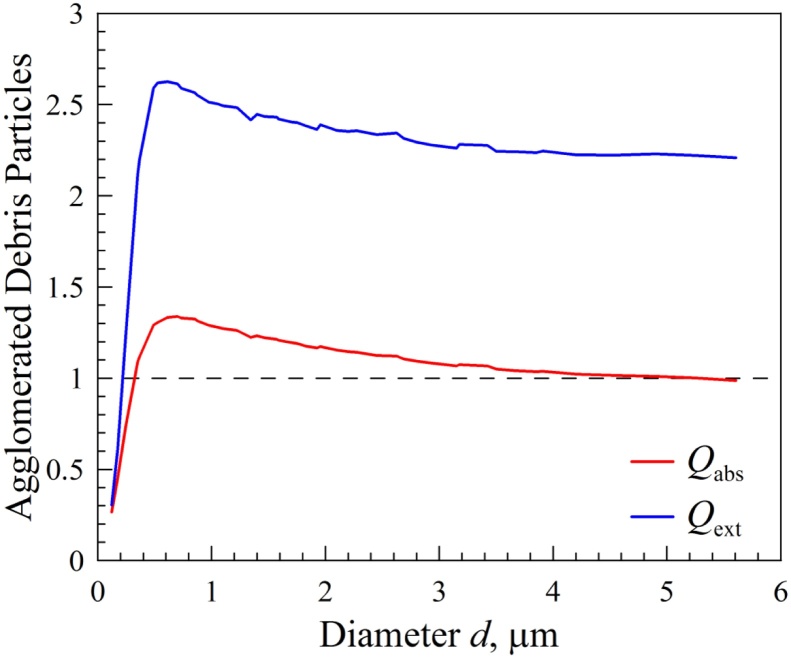
Efficiency for absorption Qabs (red line) and extinction Qext (blue line) as a function of diameter d of the agglomerated debris particles consisting of amorphous carbon (refractive index m = 2.43 + 0.59i) at green light (0.55 μm).

It is also shown that particles sized within the range of 0.5 μm to 2.5 μm are responsible for the absorption of the sun rays in the atmosphere (Fig. 3) causing this way the abnormal distribution of sunlight and associated microclimatic effects.

Two-wheeled vehicles are considered to be transportation means with high levels of environmental toxicity while emitting hazardous for the environment particulate matter consisting of great amounts of polycyclic aromatic hydrocarbons and regulated air pollutants (CO, CO_2_, HC, NO_x_, PM). The PM is able to be carried over great distances through the atmosphere [39]. In addition, the particulate matter emitted from exhaust gases is composed of soot, ash, mineral particles, and particles of a number of metals such as Cd, Cr, Cu, Fe, Hg, Ni, Pb, Se and Zn. Thus, the chemical composition of exhaust particulates makes them potentially hazardous for human health and living organisms [[40], [41]]. The presence of these toxic air pollutant particles in the ambient air has gained global attention over the last years due to their influence to environmental contamination [[4], [5], [6], [7]]. There has been reported vast amount of literature concerning these dangerous combustion products, as they exhibit toxic, mutagenic and carcinogenic properties [[11], [42], [43]] aiming to alert communities of dangerous environmental impacts. In previously conducted studies it has been proved that the toxicity of exhaust gases is increased due to the fact that the oil added to the fuel does not burn completely in the cylinder [[10], [19], [44], [45], [46]]. At last, one of the most important factors affecting the emissions of motorcycle transport is the operating life of two-wheeled vehicles. More precisely, the majority of two-wheeled vehicles used in most countries is aged 5–20 years, which makes two-wheeled transportation even more hazardous from an environmental point of view, as these vehicles do not fulfill the current technical requirements for reducing the toxic emissions [17].

In most Asian countries, two-wheeled vehicles are the main means of transportation, and therefore the pollution of the atmosphere by exhaust emissions from two-wheeled transportation means is even greater. The analysis of ambient air pollution with particulate matter of anthropogenic origin in Vietnam showed that in the regions with increased traffic of motorcycles equipped with two-stroke engines, the amount of particulate matter in the ambient air was 66%, while in regions with less traffic the amount of PM was reduced at 30% [47].

The lack of standards for the emissions of particulate matter by two-wheeled vehicles is an important issue not only in Russia and Asian countries. Up to 2013, strict regulations for the environmental control of vehicle emissions were applied for cars, freight and passenger transport, while motorcycles were under less attention on a global scale [48]. In 2013 the EU approved the Regulation No. 168/2013. In fact, this regulation sets more stringent standards for emissions of two- or three-wheeled vehicles and quadricycles. According to this document, by 2016 every two-wheeled vehicle produced and used in countries of EU should comply with the EURO 4 environmental standard, and by 2020 with the EURO 5 standard. According to this, not only emissions of CO, CH and NO_x_ are subjects of control, but also the mass of particulate matter (PM) emitted per kilometer (g km^−1^). Till today, this regulation is applied only in the European Union, while in other countries, including the Russian Federation, this regulation has not yet been adopted.

The importance of applying such regulations is evident as the results of ongoing research in this field show that the contribution of exhaust particulate matter from motorcycles to the ambient air pollution in cities is major and continues to increase. According to a research study conducted in the city of Rome, the level of contribution of exhaust emissions from motorcycles and scooters to the contamination of ambient air, was reported to be 30% [49]. Ongoing studies on air pollution in the cities of Asian countries indicate that the contribution of two-wheel transportation remains very high, while in some points of the city with limited car traffic, motorcycles are the main source of ambient air pollution [[12], [46], [50], [51]].

The reduction of the emissions of air pollutants into the atmosphere and in consequence the reductions of hazardous impacts to the environment and human health, with the introduction of more stringent standards seems to be an imperative need. For example, in China, due to the adoption of more stringent environmental standards, it is predicted that particulate emissions will be reduced by 79% by 2030 [50]. In addition, a recent EDGAR (Emissions Database for Global Atmospheric Research) analysis showed that the implementation of the EURO standards led to the reduction of particulate matter emissions in ICE exhaust by 60% worldwide [52]. At last it was shown that particles from exhaust gases are complexes of metals of carbon and various surfactants, which are capable of inhibiting the immune system of mammals [53].

Along with the increase of the number of cars and two-wheeled vehicles in the territory of the Russian Federation, the aim of reducing particulate matter in ICE exhaust emissions is the main priority. The solution for this problem lies not only in optimizing the design of engines and the exhaust systems, but also in controlling the amount of particulate matter emitted from exhaust gases of two-wheeled transportation modes by adopting new and more strict standards already applied in other countries.

## 5 Conclusions

In this research study, particle size distribution and chemical composition of particulate matter from the exhaust emissions of 44 two-wheeled vehicles is examined by laser particle size analysis and optical morphometry. In laser particle size analysis the percentage of particles within the PM_10_ fraction is much smaller than the percentage of particles obtained by optical morphometry, consisted mainly of large particles and agglomerates. As a consequence, laser particle size analysis proves to be a suitable method for the determination of particle size distribution of PM emitted from exhaust gases of motorcycles, after the agglomeration of particles. As shown with the optical morphometry method, the majority of PM originated from exhaust gases of motorcycles range within the PM_2,5_ and PM_10_ fractions. In addition there were also reported two clear assumptions. The first one is that regarding to the motorcycle brand, a few samples did not exhibit a great percentage of PM_10_ fraction. The second one is that more modern vehicles, that have a harmful gas afterburning system, are usually the source of an increased percentage of PM_10_ emitted particles. Regarding to all the above, while modern engines succeed to reduce exhaust gases, also succeed to emit particles sized under the PM_10_ fraction which are more hazardous to the environment.

At last it is shown that, particles within the range of 0,5 and 2,5 μm play a crucial role on the distribution of sunlight while also causing microclimatic effects by absorbing the sun rays.

According to the results of this study, the need for further research concerning the toxic effects of PAHs originated from exhaust emissions of two-wheeled vehicles to human health and environment is imperative.

These results will help improve our understanding of risks that the emitted particles pose to human health and environment and may strengthen the need for the adoption of new standards for the efficient control of these air pollutant particles.

## Funding sources

The work was supported by a grant from the Russian Science Foundation No. 15-14-20032.
